# The Antioxidant and Antitumor Efficiency of *Litophyton* sp. Extract in DMH-Induced Colon Cancer in Male Rats

**DOI:** 10.3390/life12101470

**Published:** 2022-09-21

**Authors:** Mahmoud Ashry, Hussam Askar, Abdallah Alian, Sabry A. H. Zidan, Doaa G. El-Sahra, Khaled G. Abdel-Wahhab, Sobhi F. Lamlom, Nader R. Abdelsalam, Mohamed E. Abd El-Hack, Heba F. Gomaa

**Affiliations:** 1 Zoology Department, Faculty of Science, Al-Azhar University, Assiut 71524, Egypt; 2 Department of Pharmacognosy, Faculty of Pharmacy, Al-Azhar University, Assiut 71524, Egypt; 3 Medical Surgical Nursing Department, Faculty of Nursing, Modern University for Technology and Information, Cairo 12055, Egypt; 4 Medical Physiology Department, National Research Centre, Giza 12622, Egypt; 5 Plant Production Department, Faculty of Agriculture (Saba Basha), Alexandria University, Alexandria 21531, Egypt; 6 Agricultural Botany Department, Faculty of Agriculture (Saba Basha), Alexandria University, Alexandria 21531, Egypt; 7 Department of Poultry, Faculty of Agriculture, Zagazig University, Zagazig 44511, Egypt; 8 Department of Zoology, Faculty of Science, Ain-Shams University, Cairo 11566, Egypt

**Keywords:** colon cancer, DMH, antitumor, *Litophyton* sp., soft coral, oxidative stress

## Abstract

One of the most common tumors to cause death worldwide is colon cancer. This study aims to investigate the antitumor potency of *Litophyton* sp. methanolic extract (LME) against DMH-induced colon cancer in adult male rats. Group (1) normal rats served as the control, group (2) normal rats were ip-injected with LME at a dose of 100 μg/kg/day, group (3) DMH-induced colon cancer animals, and group (4) colon cancer-modeled animals were treated with LME (100 μg/kg/day) for six weeks. The results revealed that injection of LME markedly regenerated the colon cancer pathophysiological disorders; this was monitored from the significant reduction in the values of serum biomarkers (CEA, CA19.9, AFP), cytokines (TNF-α and IL1β), and biochemical measurements (ALAT, ASAT, urea, creatinine, cholesterol, and triglycerides) matched significant increase of apoptotic biomarkers (CD4+); similarly, colon DNA fragmentation, MDA, and NO levels were down-regulated. In contrast, a remarkable upregulation in colon SOD, GPx, GSH, and CAT levels was noted. Moreover, the colon histopathological architecture showed obvious regenerations. Chromatography of LME resulted in the purification of two polyhydroxylated steroids (1 and 2) with potential cytotoxic activities. LME performed therapeutic potential colon tumorigenesis; therefore, LME may have a promising chemo-preventive feature against colon cancer, probably via enhancement of the apoptosis pathway, improvement of the immune response, reduction of inflammation, or/and restoration of the impaired oxidative stress.

## 1. Introduction

The second most lethal cancer in the world, colorectal cancer (CRC) will cause 10% of new cancer cases worldwide and caused 9.4% of cancer deaths in 2020. It is the third most frequent malignancy [1]. The development and progression of colorectal cancer are influenced by several risk factors, such as lifestyle choices, consumption of red or processed meat but not white meat, age, and family history [2]. Early cancer identification can save lives. However, colon cancer is frequently discovered when it is advanced and the symptoms are clear [3]. Multiple tumor suppressor genes and oncogenes undergo genetic changes that cause colorectal carcinogenesis, a multi-step process that transforms healthy colonic epithelia into metastatic cancer [4]. Additionally, colon cancer is typically a pathological result of ongoing oxidative stress, which results in DNA damage, mutations in cancer-related genes, and a cycle of cell death, mutation, and excessive creation of reactive oxygen species (ROS) and reactive nitrogen species (RNS).

Currently, nutritional supplement therapy, immunotherapy, chemotherapy, radiation therapy, and surgery are used to treat colon cancer. However, because of side effects from the treatments utilized, individual colon cancer treatment still has a low success rate. Due to their genetic instability, tumor cells quickly develop resistance to anticancer medications; as a result, it is urgently necessary to develop newer treatments with a new mechanism and increased therapeutic efficacy [2,5]. The diverse biological impacts of marine natural products are significant in the synthesis of key molecules for medication development [6]. Soft corals have a special group of metabolites that exhibit a wide range of bioactivities and structural diversity. As a result, research on marine soft corals will result in the creation of several chemically diverse compounds with a wide range of biological activities that might be applied to the pharmaceutical business [7]; there are twenty genera in the Nephtheidae family, which is a rich source of metabolites with therapeutic use [8]. The most well-known metabolites are steroids and terpenes, which are antibacterial, anti-inflammatory, and anticancer [9]. The world’s Indo-Pacific and Red Sea regions are home to the genus *Litophyton*, a well-known member of the Nephtheidae family [10]. Up to 250 bioactive substances have been produced by the genus Litophyton, most of which are polyhydroxylated steroids, sesquiterpenes, and diterpenes. It has been discovered that these secondary metabolites have intriguing biological properties, particularly in cancer treatment, where minute structural variations can significantly impact potency and selectivity [11]. This study’s major goal was to look into the anticancer, immunomodulatory, and antioxidant effects of LME against DMH-induced colon cancer in male rats.

## 2. Materials and Methods

### 2.1. Chemicals

Dimethylhydrazine (DMH) was obtained from Sigma Aldrich (St. Louis, MO, USA). JASCO P-1030 Polarimeter was used to measure the optical rotations. Bruker Avance III spectrometer at (600 and 1500 MHz) was used for ^1^H NMR and ^13^C NMR spectra, respectively, with the internal standard tetramethylsilane. HRESIMS data were recorded by a Thermo Fisher Scientific LTQ Orbitrap XL spectrometer. Diaion HP-20 (Mitsubishi Chemical Co., Ltd., Tokyo, Japan), Silica gel 60 (E. Merck, Darmstadt, Germany), Cosmosil 75C18-OPN (Nacalai Tesque, Kyoto, Japan) were used for column chromatography. HPLC analyses were performed on an Inertsil ODS-3 column (GL Science, Tokyo, Japan) monitored with a refractive index detector, RID-6A (Shimadzu, Kyoto, Japan).

### 2.2. Preparation of the Litophyton Methanolic Extract (LME)

The soft coral sample was collected from the Egyptian Red Sea coast during winter 2022, transferred to the lab immediately for the extraction process, and identified as (*Litophyton* sp.) by a specialist. Then, 1.25 kg of the sample was cut up into small pieces and macerated in methanol at room temperature. The mixture was filtered using Whatman filter paper (Merck, Darmstadt, Germany), then the solvent was evaporated using a rotary evaporator at 50 °C yielding 40 g from the 1250 g of crude coral dry powder, i.e., 3.2%, and the LME was stored at −80 °C till its chemical analysis.

### 2.3. Purification of Compounds ***1*** and ***2***

The LME was subjected to liquid–liquid fractionation as described by Mahmoud et al. [12]. First, distilled water was used to digest the LME. Next, the suspension was transferred to a separating funnel. Its components were successively divided between the aqueous layer and n-hexane (200 mL × 3), chloroform (200 mL × 3), ethyl acetate (200 mL × 3), and n-butanol (200 mL × 3). The fractions were dried under reduced pressure after the solvents from each fraction were removed, yielding the n-hexane, chloroform, ethyl acetate, n-butanol, and watery fractions. The ethyl acetate fraction was submitted to a Diaion HP-20 column (3 × 100 cm, i.d.) and successfully eluted with aqueous, methanol, and acetone. Drying these elutes under reduced pressure (at 50 °C) yielded the corresponding sub-fractions 2, 4.5, and 0.5 g. The methanol sub-fraction (4.5 g) was subjected to a silica gel CC and eluted gradients with EtOAc in n-hexane (0–100% of EtOAc) and yielded 16 fractions (M1–M16). The M15 (200 mg), eluted with 90% EtOAc, was chromatographed over reversed-phase (RP)-silica gel CC (0.5 × 25 cm, i.d) and finally purified on an RP-HPLC with MeOH–H2O, 80:20, and afforded compounds **1** and **2**.

### 2.4. Spectral Data of Compounds ***1*** and ***2***

Sarcsteroid F (1): An optically active white amorphous powder; ${\left[\alpha \right]}_{D}^{21}$-33.0 (c 0.5, CHCl3); 1H and ^13^C NMR data, see Table 1; HRESIMS *m*/*z* 489.3551 [M+ Na]+ (Calcd. for C28H50O5Na 489.3550).

24-methylenecholestane-1α, 3β, 5α, 6β, 11α-pentol-11-monoacetate (2): An optically active white amorphous powder; ${\left[\alpha \right]}_{D}^{21}$-22.0 (c 1.0, CHCl3); 1H and ^13^C NMR data, see Table 1; HRESIMS *m*/*z* 529.3503 [M+ Na]+ (Calcd. for C30H50O6Na 529.3500).

### 2.5. Animals

The National Research Centre’s animal colony in Giza, Egypt provided adult male Wistar albino rats (weighing between 170 and 200 g). For one week before the start of the experiment, the animals were housed in adequate plastic cages in a room free from any sources of chemical contamination, artificially lighted (12 h dark/light cycle), thermally regulated (25.1 °C), and humidified (50.5%). Excess tap water and standard rodent pellets were always accessible. Every animal received human care following the institutional standards for the care and use of experimental animals as determined by the NRC ethical committee.

### 2.6. Induction of Colon Cancer

Colon cancer was induced according to the method of Wang et al. [1] using DMH, which was dissolved in a buffer of 1 mM EDTA containing 1 mM sodium bicarbonate (pH 6.5). The animals were given a weekly intraperitoneal injection at a dose of 30 mg/kg body weight for 15 consecutive weeks.

### 2.7. Experimental Animals’ Design

After induction of colon cancer, both normal and colon cancer-modeled rats were rearranged randomly into four groups (8 rats each) as follows: (1) included healthy rats intraperitoneally-injected with distilled water (0.2 mL/day) as control; (2) included healthy rats ip-injected with LME (100 μg/kg/day calculated from the LD_50_ which was 1000 μg/kg) dissolved in distilled water for six weeks; (3) included colon cancer-modeled rats, and served as positive control, and (4) included colon cancer-modeled rats, which received LME therapy at a similar dose and for a similar period.

### 2.8. Blood and Tissue Sampling

All rats fasted for an additional night after the study, and blood samples were taken from them after anesthesia. The blood was then drawn into tubes without anticoagulants, allowed to clot, and centrifuged cool for 10 min at 3000 rpm to separate the sera. The sera were then divided into aliquots and stored at −80 °C until biochemical and biomarker measurements could be completed as soon as possible. Following blood collection, the rats were quickly decapitated to end their lives. The colons of each rat were then removed, cleaned in saline, and divided into two parts: one that was dried, wrapped in aluminum foil, and stored at −80 °C for the determination of oxidative stress markers, and the other that was soaked in formaldehyde-saline (10 percent) buffer for histopathological analysis.

### 2.9. Tissue Homogenization

A colon sample was homogenized in ice-cold phosphate buffer (50 mM, pH 7.4) to produce a 10 percent homogenate (*w*/*v*). The homogenate was then centrifuged for 20 min at 2600× *g* to separate the nuclear and mitochondrial fractions, and the supernatant was kept at −80 °C until the relevant measurements.

### 2.10. Biochemical Determinations

Using reagent kits purchased from Biodiagnostic, Dokki, Giza, Egypt, the following values were calculated using spectrophotometry: serum urea, creatinine, ASAT, ALAT, cholesterol, triglycerides, LDL-cholesterol, and HDL-cholesterol, as well as colon GSH, GPx, NO, CAT, and SOD values. According to Ruiz-Larnea et al. [13], the colon’s MDA level was assessed chemically.

### 2.11. DNA Fragmentation Percentage

The percentage of DNA fragmentation was evaluated as described by Perandones et al. [14]. The percentage of the fragmented DNA was calculated using the following equation:

(1) $$DNA\;fragmentation\;\%=\frac{A\;supernatant\;}{A\;supernatant+A\;pellet\;}\times 100$$

### 2.12. Biomarker, Immune Cytokines and Apoptotic Biomarker

Rat reagent ELISA-kits from SinoGeneClon Biotech Co., Hangzhou, China, were used to measure the levels of CA19.9, CEA, AFP, tumor necrosis factor-alpha (TNF-alpha), interleukin-1 beta (IL-1beta), and CD4 using the ELISA technique.

### 2.13. Histopathology

Colon specimens were processed, and paraffin sections (5 μm thick) were stained with hematoxylin and eosin [15] and investigated under a light microscope.

### 2.14. Statistical Analysis

Using a statistical analysis system (SAS) program software, the collected data were subjected to one-way ANOVA analysis, followed by Duncan’s multiple post hoc tests at a level of *p* ≤ 0.05 [16]; copyright (c) 1998 by SAS Institute Inc., Cary, NC, USA.

## 3. Results

Chromatography analysis of the LME resulted in the isolation of two polyhydroxylated sterols; the first was Sarcsteroid F, and the second was 24-methylenecholestane-1α, 3β, 5α, 6β, 11α-pentol-11-monoacetate. They were identified by comparing their spectroscopic data (1H, ^13^C NMR and HRESIMS) with the literature (Figure 1, Figure 2, Figure 3, Figure 4, Figure 5, Figure 6, Figure 7, Figure 8, Figure 9, Figure 10, Figure 11, Figure 12 and Figure 13 and Table 1).

**Table 1:** ^1^H and ^13^C NMR spectroscopic data of compounds **1** and **2**.

	1		2	
No	*δ*_H_ (*J* in H_z_)	*δ*_C_, type	*δ*_H_ (*J* in H_z_)	*δ*_C_, type
1	4.12, 1H,t (3.6)	78.1 d	3.70, 1H,t (3.6)	78.0 d
2a	2.06, 1H, m	38.1 t	2.03, 1H, m	38.9 t
2b	1.66, 1H, m		1.76, 1H, m	
3	4.21, 1H, m	64.6 d	4.19, 1H, m	64.3 d
4a	2.07, 1H, m	42.1 t	2.17, 1H, m	42.3 t
4b	1.65, 1 H, m		1.63, 1H, m	
5	-	79.4 s	-	79.3 s
6	3.34, 1H, br.s	75.5 d	3.32, 1H, br,d (2.4)	75.4 d
7a	1.73, 1H, m	36.1 t	1.13, 1H, m	35.8 t
7b	1.55, 1H, m		1.55, 1H, m	
8	1.85, 1H, m	30.7 d	1.86, 1H, m	30.2 d
9	1.89, 1H, m	48.0 d	2.27 *, 1H	45.3 d
10	-	42.1 s	-	42.9 s
11	3.92, 1H, m	67.8 d	5.12, 1 H, ddd (4.8, 10.8, 16.8)	73.1 d
12a	2.35, 1 H, dd (12.0,6.6)	51.7 t	2.27 *, 1H	47.6 t
12b	1.24, 1H, m		1.30 *, 1H	
13	-	43.2 s	-	44.1 s
14	1.19, 1H, m	56.2 d	1.30 *, 1H	56.5 d
15a	1.63, 1H, m	25.3 t	1.63, 1H, m	25.1 t
15b	1.10, 1H, m		1.14, 1H, m	
16a	1.42, 1H, m	31.6 t	2.10, 1H, m	32.0 t
16b	0.97, 1 H, m		1.88, 1H, m	
17	1.21, 1H, m	57.2 d	1.22, 1H, m	57.2 d
18	0.70, 3H, s	13.2 q	0.77, 3H, s	13.3 q
19	1.21, 3H, s	16.7 q	1. 12, 3H, s	17.6 q
20	1.37, 1H, m	37.4 d	1.42, 1H, m	36.8 d
21	0.97, 3H, d (6.6)	19.3 q	0.92, 3H, d (6.6)	19.0 q
22a	1.44, 1H, m	34.8 t	1.85, 1H, m	34.8 t
22b	0.97, 1H, m		1.50, 1H, m	
23a	1.89, 1H, m	29.3 t	1.98, 1H	29.3 t
23b	1.31, 1H, m		1.31 *, 1H,\	
24	1.20, 1H, m	40.3 d	-	157.5 s
25	1.57, 1H, m	32.7 d	2.21 *, 1H	34.8 d
26	0.82, 3H, d (6.6)	18.0 q	0.99, 3H, d (6.6)	22.3 q
27	0.87, 3H, d (6.6)	20.9 q	1.00, 3H, d (6.6)	22.4 q
28	0.81, 3H, d (6.6)	15.9 q	-	106.9 d
OAc				172.2 s
			1.99, 3H, s	21.7 q

^1^H NMR and ^13^C NMR measured in CD_3_OD [(600 MHz) and (150 MHz)]. * Multiplicities were not determined because of signals overlapping.

**Figure 14:**
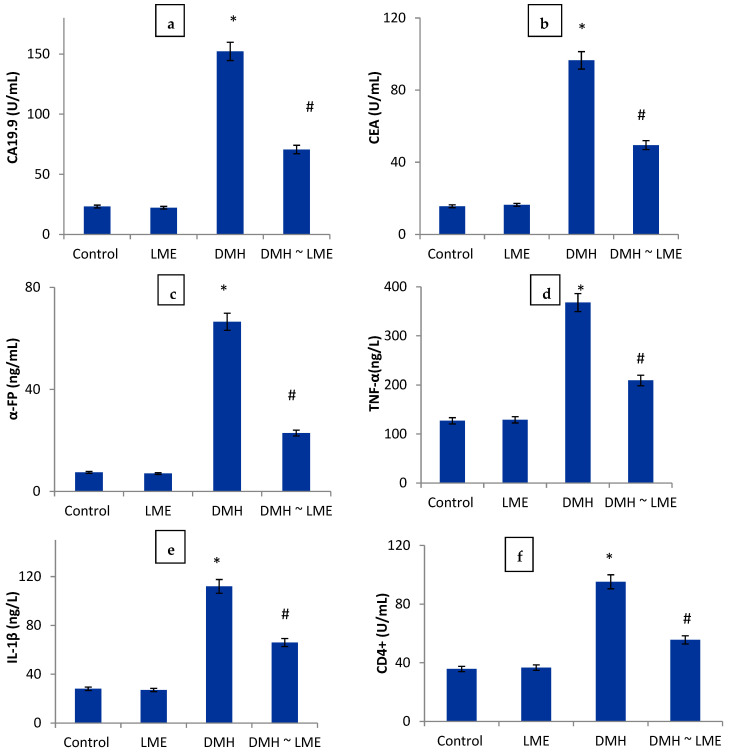

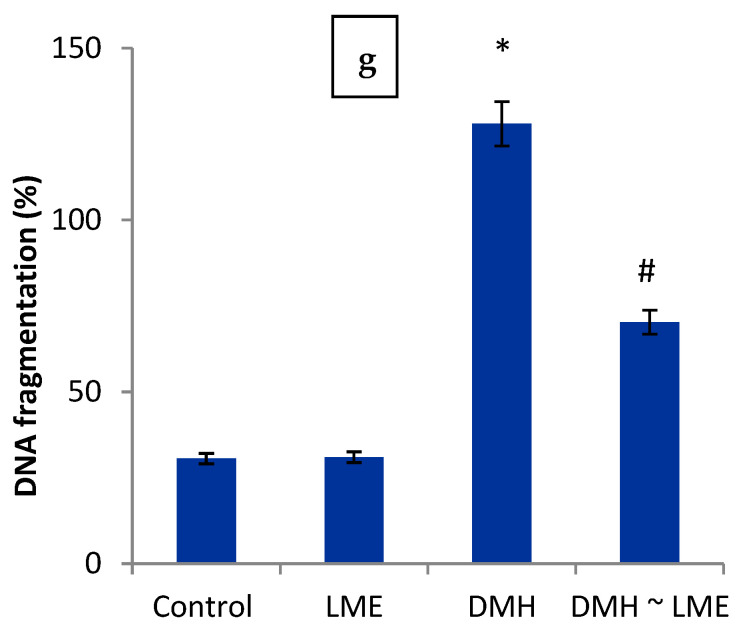
Effect of colon cancer and colon cancer-LME treated rats on serum level of (**a**) CA19.9, (**b**) CEA, (**c**) AFP, (**d**) TNF-α, (**e**) IL-1β, (**f**) CD4+, as well as (**g**) DNA fragmentation percentage. Symbol (*) is significantly different from the control group; symbol (#) is significantly different from DMH group at *p* ≤ 0.05 level; DMH is dimethylhydrazine; LME is *Litophyton* sp. methanolic extract.

The colon cancer group showed a marked increase in serum levels of CA19.9, CEA, AFP, TNF-α, IL-1β, and colon DNA-fragmentation matched with a decrease of CD4+ compared with the control group. Interestingly, treatment of colon tumor-modeled rats with LME significantly improved the levels of the mentioned biomarkers, cytokines, and apoptotic biomarkers and colon DNA fragmentation close to that of the control group (Figure 14). Additionally, colon-tumor rats showed a significant elevation in the activity of serum hepatic aminotransferases (ALAT and ASAT) and the level of kidney function markers (urea and creatinine) compared to the normal rats’ group. Injection of the colon cancer rats’ group with LME significantly ameliorated these hepatic and renal deteriorations (Table 2), favorably.

Colon cancer-affected rats’ blood lipid profiles showed atherosclerosis start, which was caused by a large increase in serum total cholesterol, triglycerides, and LDL-cholesterol, along with a noticeably lower HDL-cholesterol level. Comparatively to the group of animals used to represent colon cancer, the LME treatment of the colon cancer rat group effectively recovered lipid profile indexes (Table 3). Moreover, the rats with induced colon cancer revealed sharp disturbances in the oxidative status of colon tissues; this was evidenced via the marked drop in values of the antioxidant battery (GSH, SOD, CAT, and GPx) matched with a significant increase in the oxidative stress (MDA and NO) when compared with the control group. Fortunately, treatment of colon cancer-modeled rats with LME resulted in a significant decompensation of the depleted colon GSH content, and upregulated the activity of colon CAT, GPx, and SOD. Promisingly, LME succeeded in down-regulation of colon MDA and NO levels compared to the corresponding values of the cancered animals’ group (Table 4).

### Histopathological Examination

**Figure 15:**
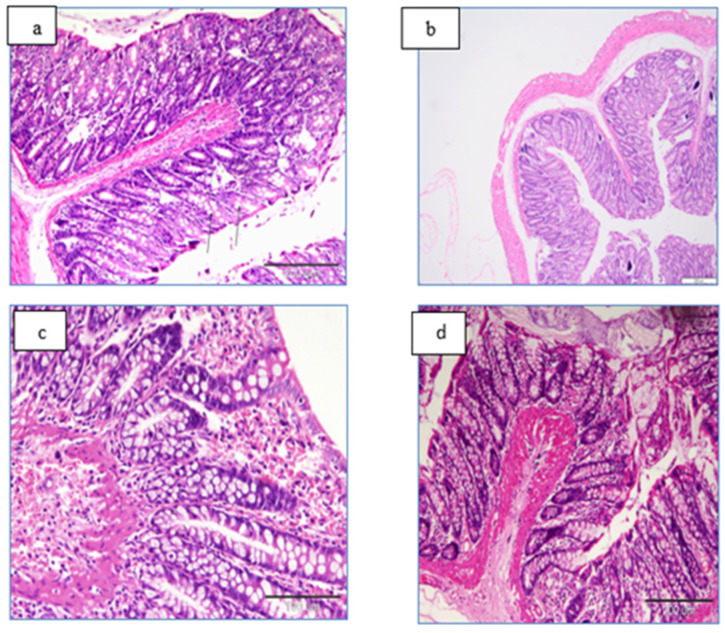
Photomicrographs of colon sections stained with H&E. (**a**) a section of normal rat colon showed normal colonic mucosa consisting of straight crypts with no villi; (**b**) a colon section of normal rat injected with LME only, showing the normal histologic structure, (**c**) a colon section of cancer modeled rats showed rupture of crypts, and huge infiltration of lymphocyte, and eosinophils were observed. In addition, adenomatous polyp was characteristic for this group; (**d**) colon section of cancer-modeled rats treated with LME showing regenerated colon architecture with just mucosal ulceration.

Light microscopic examinations of the first group (the control) and second group (the normal rat only given LME injections) showed normal colonic mucosa consisted of straight crypts with no villi (Figure 15a,b). Light microscopic observations of the third group (a colon section of cancer modeled rats) showed rupture of crypts and a huge infiltration of lymphocyte and esinophils were observed, this group was characterized by adenomatous polyps (Figure 15c). Light microscopic observations of the fourth group (cancer-modeled rats treated with LME) showed regenerated colon architecture with mucosal ulceration (Figure 15d).

## 4. Discussion

Colon cancer is the second most common form of malignancy in the world [1]; its rates are rising quickly due to inadequate treatment choices and early detection [17]. Additionally, the anticancer medications used to treat colon cancer have serious side effects that negatively influence patients’ quality of life. Therefore, effective colon cancer treatment’s main drawback is decreased susceptibility to chemotherapy with greater side effects [18]. This work was predicated on LME’s potential therapeutic and antioxidant properties as a novel anticancer drug with negligible side effects.

The increased levels of pro-inflammatory cytokines (TNF-& IL-1β), and tumor markers (CEA, CA19.9 and AFP) matched with a decrease in apoptotic biomarkers (CD4+) in colon cancer-bearing rats are in line with other earlier research [1,4,19]. Reactive oxygen species (ROS) production significantly contributes to oxidant/antioxidant imbalance. It is a well-reported component of the process behind DMH-induced colon cancer. DMH-colon cancer is primarily dependent on its biotransformation into more reactive intermediates, which takes place via two pathways: glutathione (GSH) conjugation and cytochrome P450 monooxygenases (CYPs)-dependent oxidation (phase I) (phase II) [20]; positive alterations in blood Ca19.9, CEA, and AFP levels were caused by the release of reactive oxygen species (ROS), which harm the colon and create instability in colon cell metabolism. According to the information provided, increased blood CEA and CA 19.9 are linked to either colon cancer or significantly bigger lesion sizes and numbers of adenomas [21].

The tumor microenvironment is critical to the development of cancer. An inflammatory cytokine known as tumor necrosis factor (TNF-α) is frequently present in the tumor microenvironment [22]; as a pleiotropic cytokine, TNF-α has a dual role in the development of cancer [23]; It participates in inflammation-related carcinogenesis by promoting tumor cell proliferation, survival differentiation, invasion, metastasis, and manipulation of immune responses [24]. According to numerous findings, TNF-α is elevated in various cancers, with higher levels in preneoplastic and neoplastic tissues [25], similarly to the way TNF-α-induced inflammation is exhibited by IL-1β, a pro-inflammatory cytokine [26]. Serum IL-1β levels rise in multiple investigations on cancer patients with various cancer types, showing a tumor-type independent systemic phenomenon and pointing to a relationship between disease severities in cancer patients [27].

According to a report, the metabolism of DMH leads to the development of too much ROS, which activates the p65-NF-B pathway of TNF. It may harm various proteins’ transcription and function and the advancement of epithelial cells’ transformation into invasive carcinoma in the colonic mucosa [28,29].

According to the current research, administering LME to colon cancer-model rats significantly reduced their levels of AFP, CEA, and CA 19.9, as well as immune-inflammatory markers (TNF-α and IL-1β) matched with the development of apoptotic biomarker (CD4+), compared to the control group. Numerous mechanisms, including intracellular and extracellular effects, immunoregulatory, and anti-inflammatory actions of its main ingredients, could be responsible for this impact activity, Hegazy et al. [30] The immune system has been demonstrated to be strengthened and boosted by LME [31]. Helper CD4+ T-cells play a role in adaptive immunity by conditioning the environment and modulating the activity of other immune cells through cytokine production.

The main indicators of the oxidative stress response, such as MDA and NO, were elevated in the current study, along with a decline in GSH and the related antioxidant cycle enzymes, such as GPx, SOD, and CAT, in malignant rats. Our findings revealed a substantial increase in the level of MDA in colon cancer rats, which is consistent with earlier research. Elevated MDA is an essential oxidative damage marker that has been impressively present post-DMH-intoxication [32,33]. Additionally, NO affects various cancer-related processes, such as angiogenesis, apoptosis, cell-cycle promotion, invasion, and metastasis [34]. NO may mediate nucleic acid lesions via the formation of toxicity and mutagenesis, through DNA direct degeneration or by suppressing its repair mechanisms. Cyclooxygenase is stimulated by NO, which increases the production of prostaglandins and proangiogenic substances [35]. Inducible nitric oxide synthase (iNOS), a pro-inflammatory enzyme, is expressed at high levels in colorectal cancers caused by DMH [36]. The results of the current study are supported by numerous studies that find high iNOS activity in colon cancer [37]. As a result, DNA damage has been linked to the first step in chemical carcinogenesis, and preventing DNA damage should be the first line of defense against cancer induction [38]. This suggestion coincides with the significantly increased DNA fragmentation in the rats used in the cancer model. The current study showed that LME therapy effectively reduced the MDA and NO levels of the cancerous rats and restored the antioxidant battery (SOD, GPx, CAT, and GSH) up near the control group. This beneficial improvement may be attributed to the excessive blockage and/or stabilization of free radical generation by the antioxidant potential of LME-phytochemical ingredients.

The increased levels of serum cholesterol and triglycerides observed after DMH injection are consistent with those reported by Abdel-Hamid et al. [19]. They stated that the serum triglycerides concentration is positively associated with bile acid synthesis, which may promote carcinogenesis in the large intestine. Additionally, another study suggested a positive correlation between colorectal polyps and elevated levels of total cholesterol and trigly [39]. LME demonstrated a promising antiatherogenic effect by moderating the lipogram of colon cancer rats, which was accomplished by lowering levels of cholesterol and triglycerides. This suggested that LME had the potential to inhibit the production of serum cholesterol and triglycerides, which could have an adverse effect on cell proliferation. The sarcsteroid and the 24-methylenecholestane-1, 3, 5, 6, and 11-pentol-11-monoacetate of LME may activate the enzymes responsible for cholesterol degradation and/or inhibit the cytosolic and ER enzymes involved with its formation. These processes could explain our findings. This study involved rats that were given a colon cancer model, and the rats’ serum ALAT and ASAT activities, levels of creatinine, and urea all significantly increased. This marked increase in activity may be caused by the loss of cellular functional integrity of the hepatocytes’ membrane caused by highly reactive electrophiles that severely damage the liver by inducing necrosis and fatty infiltration, methylating nucleobases, and upsetting the polysomal assembly and enzymes that are found [40]. Increased creatinine levels are a marker for renal disease [41]; In this case, the mechanism by which LME reduces the hepato-nephrotoxicity is either by lowering lipid peroxidation and changing the antioxidant defence system or by giving free radicals an electron to reduce their reactivity [42].

Regarding the histological examination, the results illustrated that rats of the control group showed normal histological features of colon architecture that matched with the finding of Alkhuriji et al. [4]; in contrast, the cancerous colon rats exhibited malignant appearances, evidenced by the elongation or oval hyperchromatic nuclei of the epithelium and glands, with the destruction of basement membrane that was similar to the finding of [43]. Destruction of the lining epithelium with leukocytic infiltrations is concurrent with [2,44] findings. It was suggested that DMH had toxic effects and acted as an inhibitor of the synthesis of nucleic acids and proteins and had carcinogenic effects in mice and rats [45]. Additionally, DMH is a pro-carcinogen that, following metabolic activation, causes the production of O6-MeG in the DNA of the colon and causes cancers in the colorectum [46].

Treatment of colon cancerous rats with LME resulted favorably in a clear regeneration of the DMH-deteriorated colon histological structures, achieving the disappearance of most malignant features.

The chemical and biological characteristics of L. *arboreum* have only been the subject of several studies [9,42,47]. Sesquiterpenes, diterpenes, and polyhydroxylated steroids are just a few of the 250 bioactive secondary metabolites that *Litophyton* has been found to produce. These substances have been found to have therapeutic biological effects on cancer management, where small structural changes can affect potency and selectivity [11].

## 5. Conclusions

In conclusion, the present study indicates that the methanolic extract of the *Litophyton* sp. of coral (LME) performed anti-colon cancer therapeutic potential. This effect was achieved from the marked improvement in the biomarkers, immunoinflammatory, oxidative status, and histopathological results. It may be mechanized through the induction of apoptosis by activation of caspases and/or CD4+ cells. It can be suggested that LME may serve as a strong potential drug for the treatment and possible prevention of cancer.

## Figures and Tables

**Figure 1:**
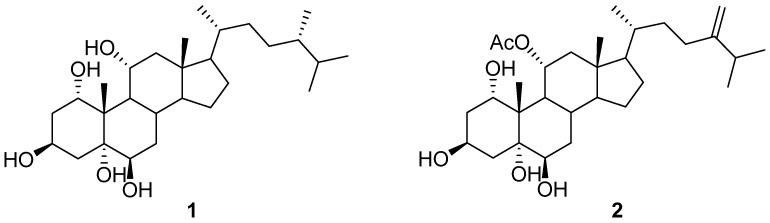
Sarcsteroid F (**1**) and 24-methylenecholestane-1α, 3β, 5α, 6βe, 11α-pentol-11-monoacetate (**2**) isolated from the LME.

**Figure 2:**
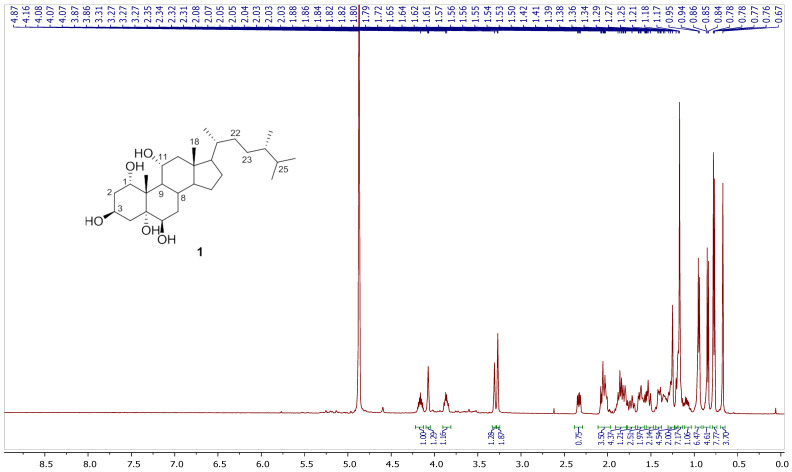
^1^H NMR spectrum of compound **1** measured in CD_3_OD (600 MHz).

**Figure 3:**
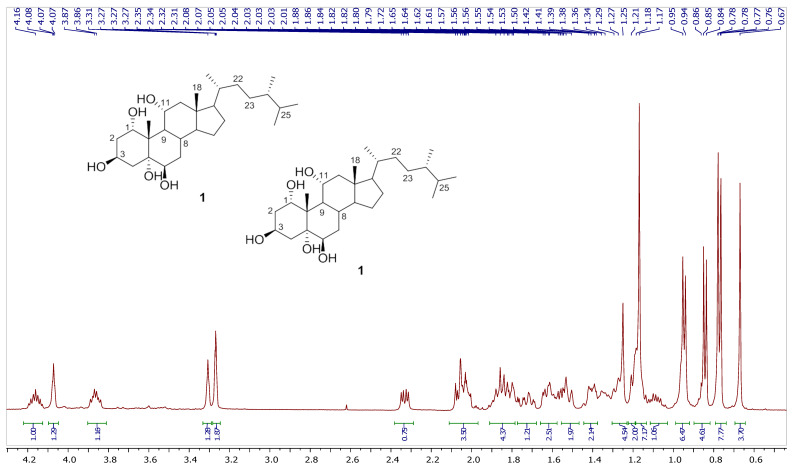
Expanded ^1^H NMR spectrum of compound **1** measured in CD_3_OD (600 MHz).

**Figure 4:**
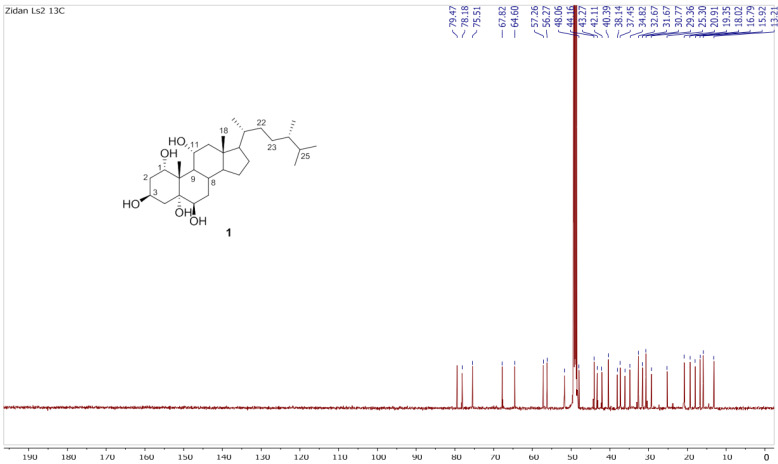
^13^C NMR spectrum of compound **1** measured in CD_3_OD (150 MHz).

**Figure 5:**
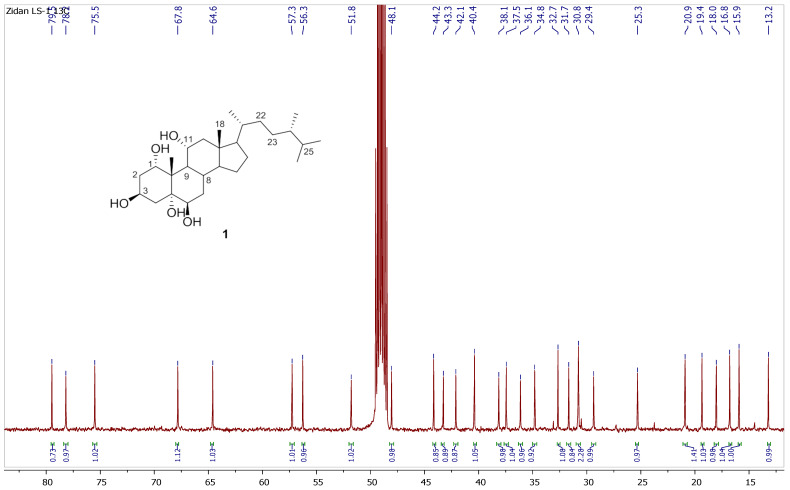
Expanded ^13^C NMR spectrum of compound **1** measured in CD_3_OD (150 MHz).

**Figure 6:**
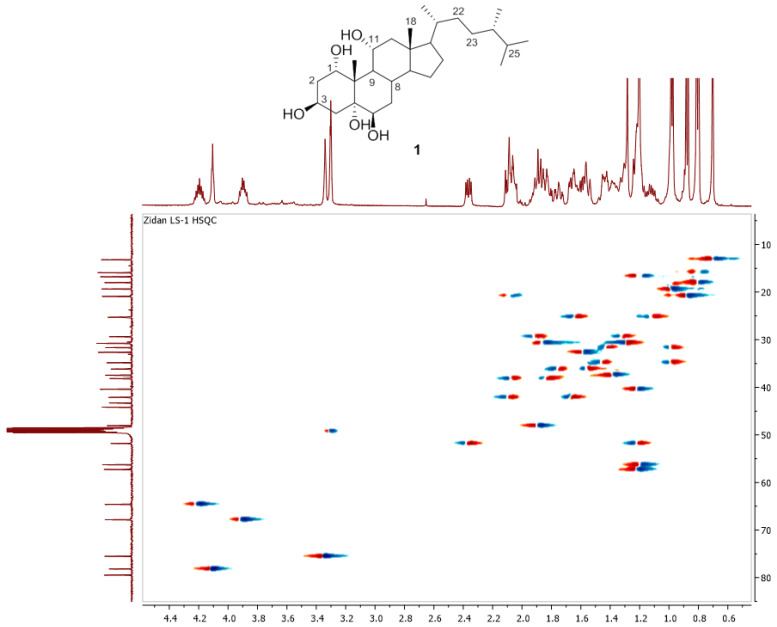
HSQC spectrum of compound **1** measured in CD3OD (150 MHz).

**Figure 7:**
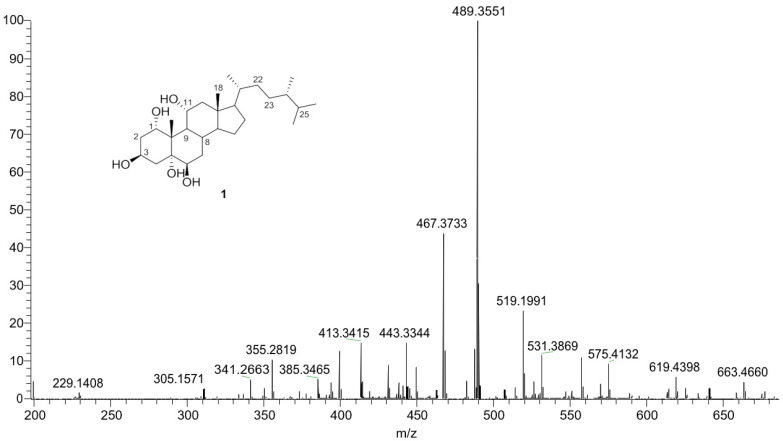
HRESIMS spectrum of compound **1**.

**Figure 8:**
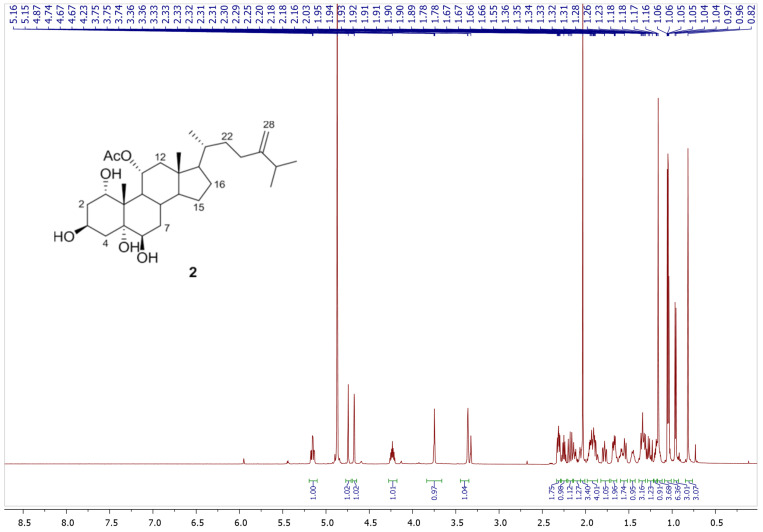
^1^H NMR spectrum of compound **2** measured in CD_3_OD (600 MHz).

**Figure 9:**
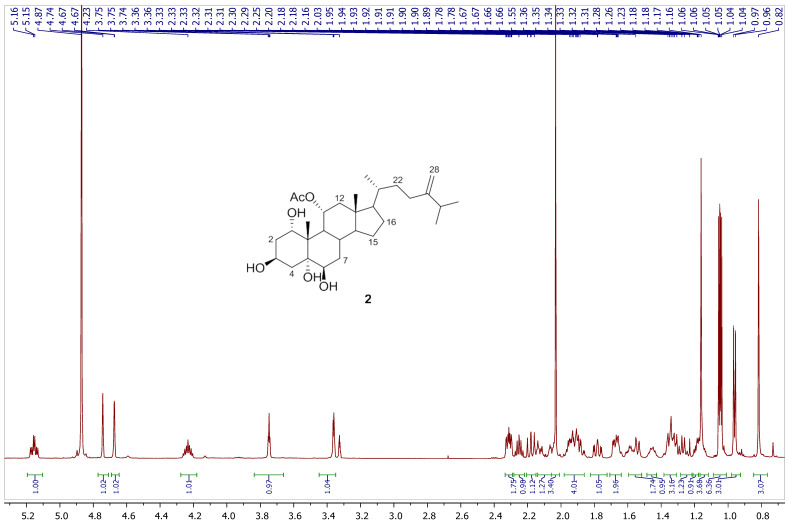
Expanded ^1^H NMR spectrum of compound **2** measured in CD_3_OD (600 MHz).

**Figure 10:**
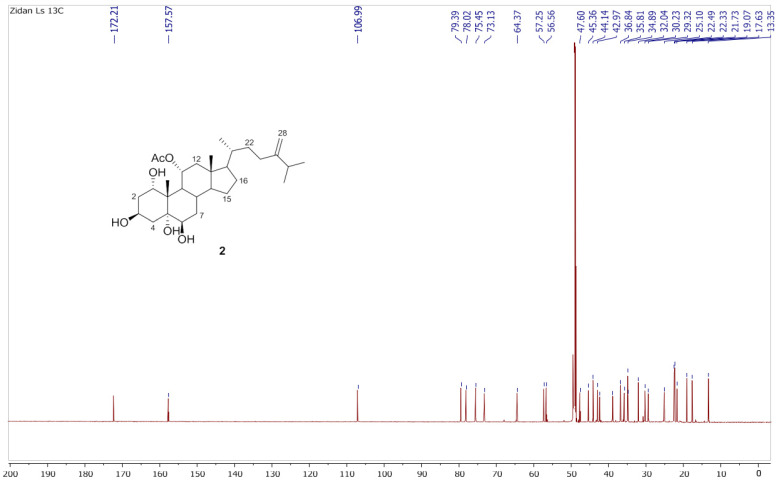
^13^C NMR spectrum of compound **2** measured in CD_3_OD (150 MHz).

**Figure 11:**
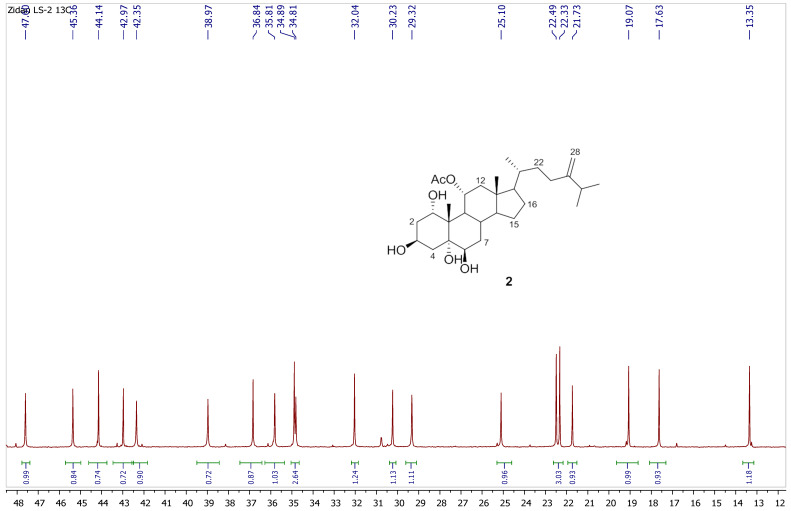
Expanded ^13^C NMR spectrum of compound **2** measured in CD_3_OD (150 MHz).

**Figure 12:**
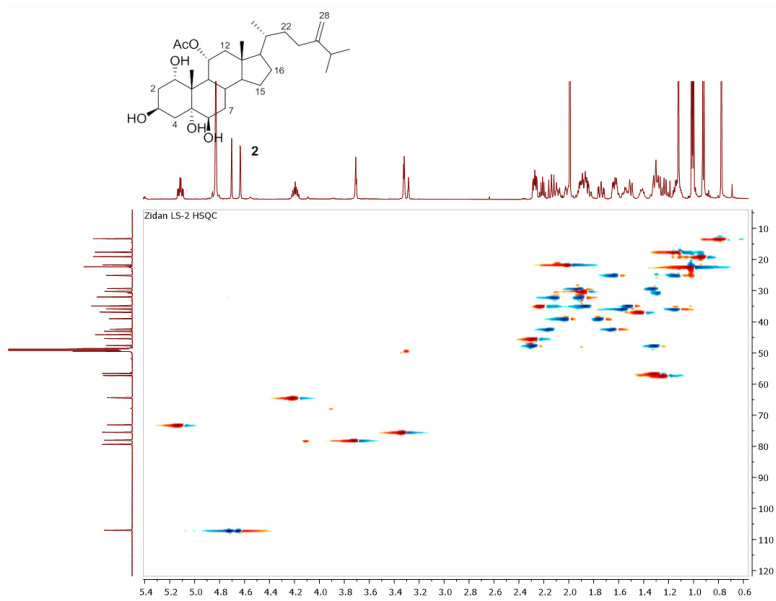
HSQC spectrum of compound **2** measured in CD_3_OD (150 MHz).

**Figure 13:**
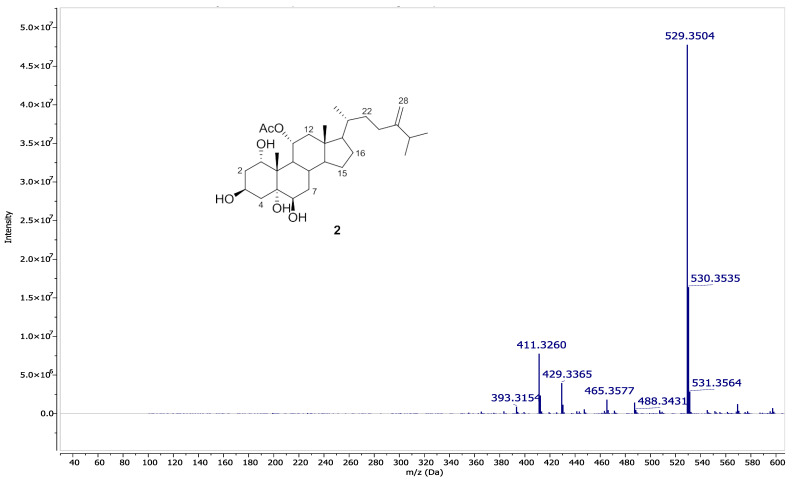
HRESIMS spectrum of compound **2**.

**Table 2:** Serum liver and kidney functions of normal, colon cancer and colon cancer-treated rats.

	Control	LME	DMH	DMH~LME
ALAT (U/L)	59.2 ± 7.1	57.8 ± 5.1	109.2 ± 6.1 *	67.6 ± 5.4 #
ASAT (U/L)	73.2 ± 3.7	71.9 ± 4.9	141.5 ± 10.9 *	80.9 ± 9.1 #
Creatinine (mg/dL)	0.91 ± 4.1	0.89 ± 2.1	1.67 ± 3.9 *	1.16 ± 5.1 #
Urea (mg/dL)	45.2 ± 3.2	44.2 ± 2.9	60.3 ± 4.1 *	49.4 ± 2.2 #

Data are presented as mean ±standard error of mean; data were subjected to one-way ANOVA fol lowed by post hoc (Tukey) test at *p* ≤ 0.05. Symbol (*) is significantly different from the control group; symbol (#) is significantly different from DMH group at *p* ≤ 0.05 level; DMH is dimethylhydrazine; LME is *Litophyton* sp. methanolic extract.

**Table 3:** Serum lipid profile of normal, colon cancer, and colon cancer-treated rats’ groups.

	Control	LME	DMH	DMH~LME
CHO (mg/dL)	140 ± 2.5	135.1 ± 6.1	225.3 ± 10.5 *	171.4 ± 5.66 #
TRG (mg/dL)	120.1 ± 4.1	123.3 ± 4.44	215.5 ± 9.4 *	151.9 ± 6.99 #
HDL (mg/dL)	44.1 ± 1.01	45.01 ± 3.9	34.1 ± 1.66 *	42.1 ± 4.8 #
LDL (mg/dL)	72.1 ± 3.5	65.4 ± 2.01	148.5 ± 3.2 *	98.8 ± 2.88 #

Data are presented as mean ±standard error of mean; data were subjected to one-way ANOVA followed by a post hoc (Tukey) test at *p* ≤ 0.05. Symbol (*) is significantly different from the control group; symbol (#) is significantly different from DMH group at *p* ≤ 0.05 level; DMH is dimethyl hydrazine; LME is *Litophyton* sp. methanolic extract.

**Table 4:** Colon oxidant–antioxidant markers of control, colon cancer, and colon cancer-treated rats’ groups.

	Control	LME	DMH	DMH~LME
MDA (μmol/g)	6.2 ± 0.33	6.01 ± 0.54	18.4 ± 1.22 *	9.45 ± 1.02 #
NO (μmol/g)	80.3 ± 8.9	78.5 ± 10.2	156.4 ± 15.4 *	92.5 ± 6.8 #
GSH (nmol/g)	252 ± 25.6	261 ± 24.5	110.5 ± 18.55 *	221.4 ± 15.4 #
SOD (U/g)	375 ± 16.5	386 ± 31.2	164.5 ± 12.4 *	295 ± 18.4 #
GPx (U/g)	305 ± 14.2	302 ± 24.1	130.4 ± 16.5 *	245 ± 21.6 #
CAT(U/g)	15.4 ± 0.94	16.4 ± 0.65	7.55 ± 1.1 *	11.6 ± 2.1 #

Data are presented as mean ±standard error of mean; data were subjected to one-way ANOVA followed by post hoc (Tukey) test at *p* ≤ 0.05. Symbol (*) is significantly different from the control group; symbol (#) is significantly different from DMH group at *p* ≤ 0.05 level; DMH is dimethylhydrazine; LME is *Litophyton* sp. methanolic extract.

## Data Availability

Not applicable.
